# Incidences of *Rhipicephalus* (*Boophilus*) *microplus* (Canestrini, 1888) Transmitted Pathogens in Cattle in West Africa

**DOI:** 10.1007/s11686-022-00568-8

**Published:** 2022-06-17

**Authors:** Alassane Toure, Moussa Sanogo, Abdelmalek Sghiri, Hamid Sahibi

**Affiliations:** 1 LANADA/Laboratoire Central Vétérinaire de Bingerville, Po Box: 206, Bingerville, Côte d’Ivoire; 2 Institut Agronomique et Vétérinaire Hassan II, Madinat Al Irfane, Po Box: 6202, Rabat, Morocco

**Keywords:** Incidence density, Babesiosis, Anaplasmosis, PCR, Côte d’Ivoire

## Abstract

**Context and Purpose:**

In a context of recent introduction of *Rhipicephalus* (*Boophilus*) *microplus* tick species in West Africa, the purpose of the authors is to estimate incidence density of cattle babesiosis either caused by *Babesia bigemina* or *Babesia bovis*, and cattle anaplasmosis. Incidence density represents how quickly a disease or a condition is occurring amongst a group of individuals at risk.

**Methods:**

The longitudinal and prospective study design took place in south, centre, east, west and north of Côte d’Ivoire. Cattle have been followed for 12 months. At the end of each month, each animal has been RT-PCR tested for new infection by *Babesia bovis*, *Babesia bigemina*, and PCR–RFLP tested for new infection by *Anaplasma marginale*.

**Results:**

Findings show for the study area that incidence densities of *Babesia bovis*, *Babesia bigemina* and *Anaplasma marginale* infections in Côte d’Ivoire are, respectively, 15.3 new infections [95% CI 13.1–17.88] per 100 cattle, 32.2 new infections [95% CI 28.5–36.3] per 100 cattle, and 25.9 new infections [95% CI 22.5–29.6] per 100 cattle.

**Conclusion:**

Finally, there is increasing of infection incidence density following the region distance from the coast or elevation.

## Introduction

Tick-borne diseases are known to cause major impacts on livestock and economy particularly in Africa [1, 2]. In West African countries (WAC), contribution of livestock sector in the Gross National Product is at least 20% and contributes more than 52.2% of people employment [3]. Moreover 60% of west African population or more than 226 million persons depend on this activity in 2018 [4]. Therefore, it is crucial to reduce the constraints on the livestock such as tick-borne diseases. At national or regional level, there is a lack of continuing surveillance of tick-borne diseases. In this condition, incidence, which is one of the essential epidemiological parameters related to the risk, is rarely accurately estimated. The incidence rate is a measure of the frequency with which a disease or other incident occurs over a specified time period; when the denominator is the sum of the animal-time of the at-risk population, it is also known as the incidence density or also called animal-time incidence rate [5]. This procedure implies to rigorously check new infection not only in each subject participating to the study but also in a predefined constant duration of time. In consequence, this context makes difficult the design of short, medium or long-term preventive or control strategies. In addition, soon after 2000 in West Africa, uncontrolled living cattle importation from Brazil led presumably to introduction and efficient spread of a new tick species: *Rhipicephalus* (*Boophilus*) *microplus* [6]. In cattle, this tick is an efficient vector of *Babesia bovis*, *Babesia bigemina*, and *Anaplasma marginale*. These pathogens are, respectively, the aetiology of cattle babesiosis and cattle anaplasmosis. Besides animal health concerns, these diseases cause several 10 million of dollar losses [2, 7]. This introduction and spread event could modify dynamic of diseases occurrence in WAC, justifying the need of updated epidemiological parameters. The main objective of this study is to estimate incidence density of cattle babesiosis due to *Babesia bigemina* and *Babesia bovis*, and cattle anaplasmosis caused by *Anaplasma marginale* in the first WAC of *Rhipicephalus* (*Boophilus*) *microplus* tick vector introduction, *i.e*. Côte d’Ivoire.

## Materials and Methods

### Study Area and Blood Collection

Our study has been conducted in south, centre, east, west, and north of Côte d’Ivoire. These regions are part of the WAC of introduction of the tick *Rhipicephalus* (*Boophilus*) *microplus*. Each of the city selected for the current study in Côte d’Ivoire (north: Odienné (elevation 437 m**)**, Korhogo (elevation 380 m), Ferkessedougou (elevation 316 m); west: Man (elevation 240 m**)**, east: Agnibilekrou (elevation 190 m); centre: Bouaké (elevation 363 m), Yamoussoukro (elevation 200 m**)**; south: Bingerville (elevation 10 m), Dabou (elevation 15 m)) is owing to the first reports of *Rhipicephalus* (*Boophilus*) *microplus* and its spreading to the rest of the country [6, 8].

A longitudinal study was carried out between March 2013 and March 2014. Before including in the study, each cattle has been molecular negative tested. Monthly blood samples were collected from each cattle. There were no preventive measures against babesiosis and anaplasmosis during the study. In case of diseases, diagnosis is made followed by gracious veterinary cares. At each site or town, 12 cattle over 1 year old were randomly included in the study to detect new infections with *Babesia bigemina*, *Babesia bovis*, and *Anaplasma marginale* each month. A total of 12 visits were made and 144 samples collected per site or town.

### Molecular Detection of Pathogens

The diagnosis of babesiosis was made by looking for the presence of *B. bovis* and *B. bigemina* in the samples by Real Time PCR (RT-PCR) [9]. A multiplex mix containing both the primers of *B. bovis* (cbosg-1 and cbosg-2) and *B. bigemina* (cbisg-1 and cbis-2) was validated by a team of CIRAD-EMVT Guadeloupe and was used during this study. The detection of *A. marginale* by nested RFLP-PCR [10, 11], was made by targeting the Msp5 genes [12] and Msp4 [13]. The primers used for detections are reported (Table 1).

**Table 1:** Details of primers used for PCR testing targeting *A*. *marginale, Babesia bovis* and *Babesia bigemina*

Tests	Primers	Sequences	Expected sizes
RT-PCR	*B. bovis*: cbosg-1	5′TGTTCCTGGAAGCGTTGATTC3′	88 bp
	*B. bovis*: cbosg-2	5′AGCGTGAAAATAACGCATTGC3′	
	*B. bigemina*: cbisg-1	5′TGTTCCAGGAGATGTTGATTC3′	
	*B. bigemina*: cbisg-2	5′AGCATGGAAATAACGAAGTGC3′	
Semi-nested PCR (Msp5)	Msp5extFor	5′GCATAGCCTCCGCGTCTTTC3’	Round 1: 456 bp Round 2: 343 bp
	Msp5intFor	5′TACACGTGCCCTACCGAGTTA3′	
	Msp5extRev	5′TCCTCGCCTTGGCCCTCAGA3′	
Conventional PCR (Msp4) + RFLP (TaqI)	Msp45	5′GGGAGCTCCTATGAATTACAGAGAATTGTTAC3′	*A. marginale* and *A. ovis*: 866 bp
	Msp43	3′CCCCGGATCCTTAGCTGAACAGGAATCTTG5′	Digestion *A. marginale*: 198, 141 and 527 bp

### Statistical Data Analysis

The data were entered with the Excel 2010 software. The data was coded and analysed to estimate the incidence density. The Chi-square statistical test, the one-way analysis of variance, the correlations between the different incidences at cattle population level and according to the regions were determined. The dependence of incidence density of different diseases on the region was verified according to a generalised linear model (glm). The model used in this study is a linear model with the categorical variables represented by the different regions. The estimates were performed using the software R-3.3.3.

## Results

### Incidence Density of Diseases According to the Different Study Areas

#### Bovine Babesiosis Caused by *Babesia bovis*

**Fig. 1:**
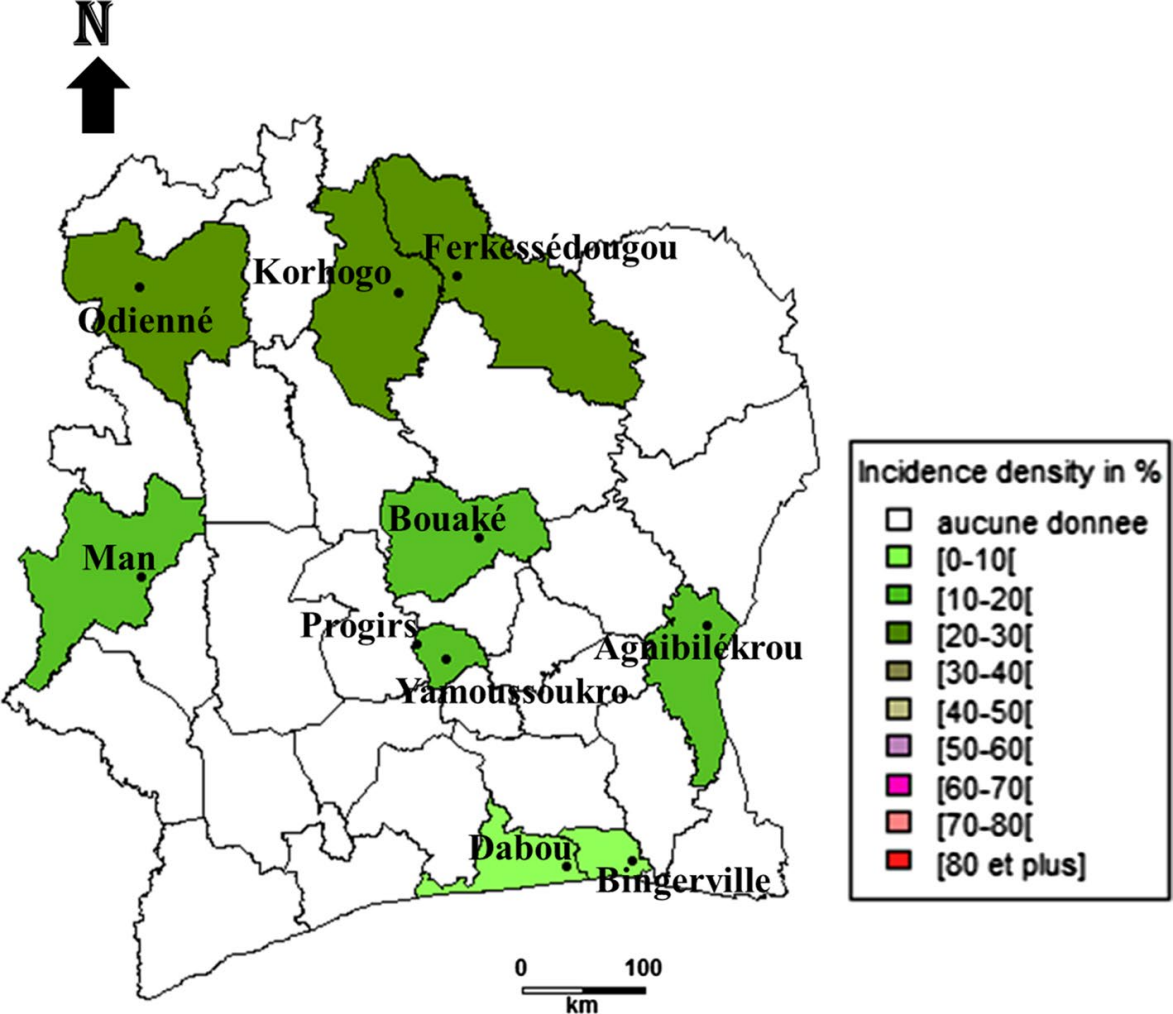
Incidence density of *Babesia bovis* cattle infection in different regions of Côte d’Ivoire

Incidence density of *Babesia bovis* infection varies from 2.36 new infections [95% CI 0.6–6.43] per 100 cattle in the east region to 40.17 [95% CI 32.5–49.2] new infections per 100 cattle in the region of north. The south region also has a low incidence density of 4.31 new infections [95% CI 1.75–8.98] per 100 cattle. The centre region and the west ones have respective incidence density of 11.30 new infections [95% CI 8.4–14.9] per 100 cattle and 9.92 new infections [95% CI 5.65–16.26] per 100 cattle (Fig. 1).

#### Bovine Babesiosis Caused by *Babesia bigemina*

**Fig. 2:**
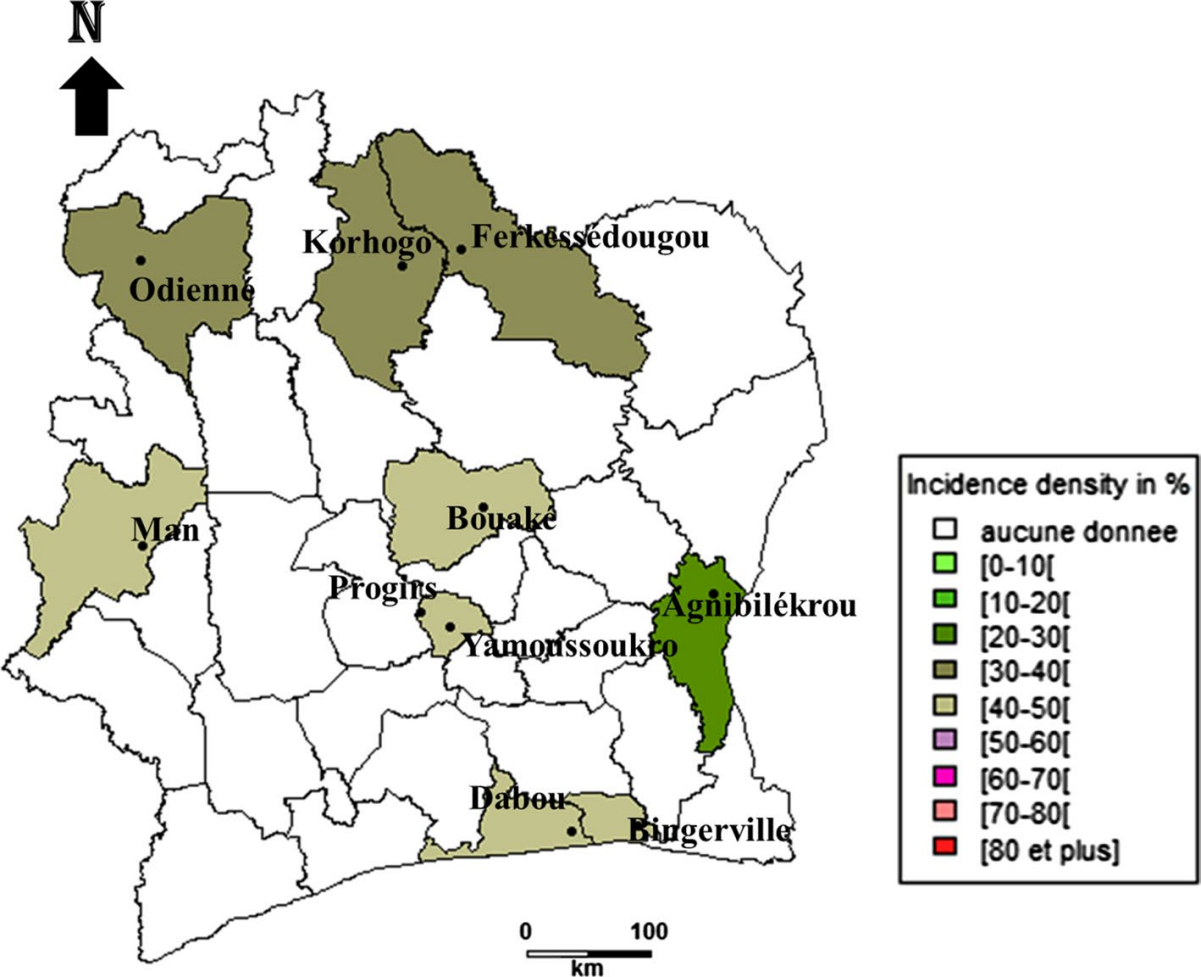
Incidence density of *Babesia bigemina* cattle infection in different regions of Côte d’Ivoire

The incidence density of bovine babesiosis caused by *Babesia bigemina* show relatively high values in all the regions studied. The minimum value is reached in the east with 21.80 new infections [95% CI 14.88–30.91] per 100 cattle, and a maximum value of 56.9 new infections [95% CI 38.8–80.6] per 100 cattle in the north region. The west, south and centre regions have incidences of 31 new infections [95% CI 23.97–39.48] per 100 cattle, 23.5 new infections [95% CI 16.25–32.95] per 100 cattle and 37.2 new infections [95% CI 30.8–44.6%] per 100 cattle, respectively (Fig. 2).

#### Anaplasmosis Caused by *Anaplasma marginale*

**Fig. 3:**
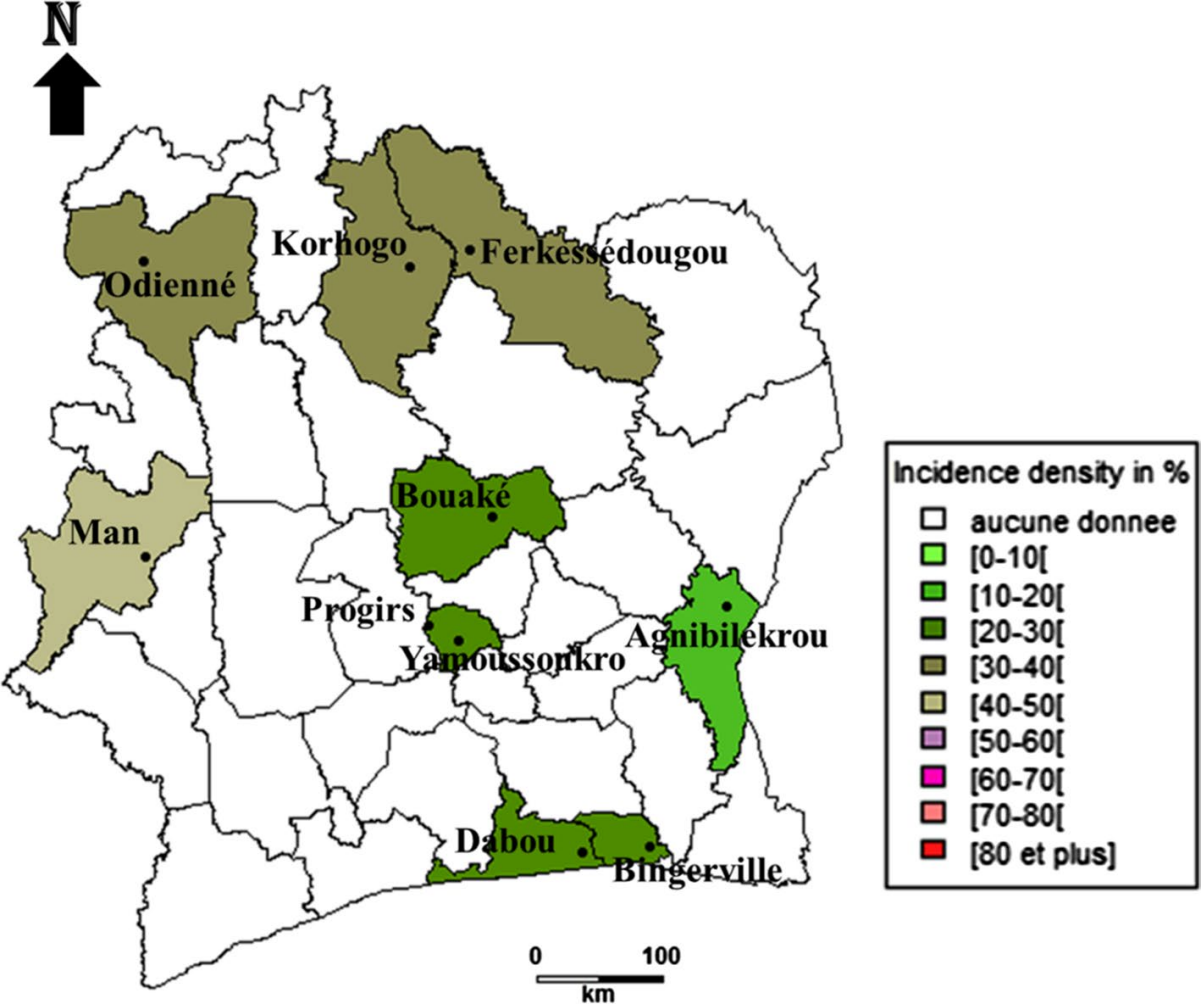
Incidence density of *Anaplasma marginale* cattle infection in different regions of Côte d’Ivoire

Concerning incidence density of bovine anaplasmosis caused by *Anaplasma marginale*, it shows the highest values in the north and west regions with 47.9 new infections [95% CI 33.92–65.95] per 100 cattle and 47.4 new infections [95% CI 33.7–65.9] per 100 cattle, respectively. These regions are followed by centre with 33.8 new infections [95% CI 27.8–40.7] per 100 cattle. The lowest values are obtained in the south and east regions with respective values of 12.4 new infections [95% CI 8.3–17.9] per 100 cattle and 2.5 new infections [95% CI 0.28–5.6] per 100 cattle (Fig. 3).

### Trend of the Incidence Density Variations

The incidence density of *Babesia bovis* infection in the total population studied is 15.3 new infections [95% CI 13.1–17.88] per 100 cattle with 1046 bovines-months. The incidence density of this infection has a significant regional effect (*p* < 0.0001). In fact, the incidence density of the disease was 4.3 new infections [95% CI 1.75–8.98] per 100 cattle in the south region, 2.4 new infections [95% CI 0.6–6.43] per 100 cattle in the east region, 9.9 new infections [95% CI 5.65–16.26] per 100 cattle and 11.30 new infections [95% CI 8.4–14.9] per 100 cattle in the west and centre regions, respectively. Finally, as a whole, from the lowest incidence density in the south, then increased values in centre and west, incidence density reaches highest value in the north region with 40.18 new infections [95% CI 32.5–49.2] per 100 cattle (Table 2).

**Table 2:** Results concerning impact of region on incidence: cases of *Babesia bovis Babesia bigemina and Anaplasma marginale* cattle infections

Pathogens	Region	Bovines-months	Number of new cases (new infections per 100 bovines)	95% confidence interval (%)	Chi^2^
*Babesia bovis*	South	139	6 (4.32)	(0.94–7.70)	95.1374
	East	127	3 (2.36)	(0.0–5.00)	
	West	141	14 (9.93)	(5.00–14.86)	
	North	224	90 (40.18)	(33.75–46.60)	
	Centre	416	47 (11.30)	(8.26–4.34)	
	Total	1046	160 (15.3)	(13.11–17.48)	
*Babesia bigemina*	South	132	31 (23.48)	(16.25–30.71)	14.107
	North	51	29 (56.86)	(43.27–70.45)	
	East	133	29 (21.80)	(14.79–28.82)	
	West	200	62 (31)	(24.59–37.41)	
	Centre	301	112 (37.2)	(30.78–41.48)	
	Total	817	263 (32.19)	(28.98–35.39)	
*Anaplasma marginale*	North	73	35 (47.94)	(39.42–56.47)	63.436
	East	118	2 (1.70)	(0.00–4.02)	
	West	76	36 (47.37)	(36.14–58.59)	
	South	210	26 (12.38)	(7.82–16.94)	
	Centre	311	105 (33.76)	(28.60–39.14)	
	Total	788	204 (25.88)	(24.05–30.26	

*P* value *Babesia bovis*. < 0.0001, *P* value *Babesia bigemina* < 0.007, *P* value *Anaplasma marginale*. < 0.0001

As for the incidence density of *Babesia bigemina* infection in the general population, it is 32.2 new infections [95% CI 28.5–36.3] per 100 cattle with 817 bovines-months. The incidence densities in the south and north regions are, respectively, 23.5 new infections [95% CI 16.25–32.95] per 100 cattle and 56.9 new infections [95% CI 38.8–80.6] per 100 cattle. In addition, the regions of east, west and centre have incidence densities of 21.80 new infections [95 CI 14.88–30.91] per 100 cattle, 31 new infections [95% CI 23.97–39.48] per 100 cattle and 32.50 new infections [95% CI 30.8–44.6] per 100 cattle, respectively. The region has a very significant effect on incidence densities (*p* = 0.007) by depicting a trend of lowest value in south, followed by moderate increasing in the west and centre, excepted for east, to the highest value in north (Table 2).

Regarding the incidence density of *Anaplasma marginale* infection in the same population, it is 25.9 new infections [95% CI 22.5–29.6] per 100 cattle with 788 bovines-months. The highest value of 47.9 new infections [95% CI 33.92–65.95] per 100 cattle is observed in the north region, followed by the west region with 47.4 new infections [95% CI 33.7–65.9] per 100 cattle. The regions of east, south and centre have incidence densities of 1.70 new infections [95% CI 0.28–5.6] per 100 cattle, 12.4 new infections [95% CI 8.3–17.9] per 100 cattle and 33.8 new infections [95% CI 27.8–40.7] per 100 cattle, respectively. There is also a very significant effect of the regions on incidence density (*p* < 0.0001) (Table 2) with a global and increasing trend from south to the north.

### Incidence Density Variation of *Babesia bovis*, *Babesia bigemina *and *Anaplasma marginale* Infections Comparing to North Region

Whatever the distance from north region and the infection considered, there is a very significant effect on the incidence density, namely Babesiosis due to *Babesia bovis* (*p* = 2.10^–16^) (Table 4), or due to *Babesia bigemina* (*p* < 0.001) (Table 5), and Anaplasmosis caused by *Anaplasma marginale* (*p* < 0.0001) (Table 3).

**Table 3:** ANOVA one-way concerning the effect of the region on the incidence of *Babesia bovis, Babesia bigemina,* and *Anaplasma marginale* infections

Pathogens		Df	Sum Sq	Mean Sq	*F* value	Pr (> F)	Significance
*Babesia bovis*	Region	4	1.26860203	0.317150509552008	17.99160	1.6916422919e-09	***
	Residuals	55	0.9695232	0.0176276948089607			
*Babesia bigemina*	Region	4	1.08943485590975	0.272358713977438	6.8057123555812	0.000159297	***
	Residuals	55	2.20105236397107	0.0400191338903831			
*Anaplasma marginale*	Region	4	1.87945556916485	0.469863892291212	8.15179462695865	3.09543866341821e-05	***
	Residuals	55	3.17016255421271	0.0576393191675038			

0 ‘***’ 0.001 ‘**’ 0.01 ‘*’ 0.05 ‘.’ 0.1 ‘’ 1

In particular for *Babesia bovis* infection, the incidence density in each of the regions tends to decrease significantly compared to that of the north region. They are below − 0.40503 ± 0.05420 for west to − 0.31791 ± 0.05420 for the east (Table 4).

**Table 4:** Results of generalised linear model concerning incidence density variation of *Babesia bovis* infection comparing to north region

	Estimate	Std. error	*t* value	Pr ( >|t|)	Significance
(Intercept)	0.4265	0.03833	11.128	1.06e-15	***
Centre	− 0.3479	0.05420	− 5.808	3.28e-07	***
East	− 0.40503	0.05420	− 7.473	6.34e-10	***
West	− 0.31791	0.05420	− 5.865	2.65e-07	***
South	− 0.37523	0.05420	− 6.923	5.05e-09	***

Signif. codes: 0 ‘***’ 0.001 ‘**’ 0.01 ‘*’ 0.05 ‘.’ 0.1 ‘’ 1. Residual standard error: 0.1065 on 55 degrees of freedom, multiple R-squared: 0.5796, Adjusted R-squared: 0.549, F-statistic: 18.96 on 4 and 55 DF, *p* value: 7.581e-10

In terms of the effect of different regions on the incidence density of *Babesia bigemina* infection, the sole incidence density significant decreasing is observed in the west region compared to the north one (*p* < 0.001). The decreasing is not significant for the south region. It should also be noted that the incidence densities of the centre and the east regions tend to be significantly close to zero (Table 5).

**Table 5:** Results of generalised linear model concerning incidence density variation of *Babesia bigemina* infection comparing to north region

	Estimate	Std. error	*t* value	Pr ( >|t|)	Significance
(Intercept)	0.30852	0.05775	5.343	1.81e-06	***
Centre	0.05196	0.08167	0.636	0.52727	
West	0.27263	0.08167	3.338	0.00152	***
East	− 0.09464	0.08167	− 1.159	0.25156	
South	0.09262	0.08167	− 1.134	0.26170	

0 ‘***’ 0.001 ‘**’ 0.01 ‘*’ 0.05 ‘.’ 0.1 ‘’ 1. Residual standard error: 0.1854 on 55 degrees of freedom, multiple R-squared: 0.443, Adjusted R-squared: 0.4025, F-statistic: 10.94 on 4 and 55 DF, p-value: 1.352e-06

With regard to the incidence densities of *Anaplasma marginale* infection, in the south, west and centre regions, they are down compared to the north region (*p* < 0.001; *p* < 0.01 and *p* < 0.01). Incidence densities decreased from − 0.33215 ± 0.09801 for south region to 0.21548 ± 0.09801 for the centre one (Table 6).

**Table 6:** Results of generalised linear model concerning incidence density variation of *Anaplasma marginale* infection comparing to north region

	Estimate	Std. error	*t* value	Pr ( >|t|)	Significance
(Intercept)	0.13193	0.06931	1.904	0.06219	
Centre	0.21548	0.09801	2.199	0.03214	*
East	− 0.10087	0.09801	− 1.029	0.30790	
West	− 0.33414	0.09801	3.409	0.00123	**
South	− 0.33215	0.09801	− 8.233	3.64e-11	***

0 ‘***’ 0.001 ‘**’ 0.01 ‘*’ 0.05 ‘.’ 0.1 ‘’ 1. Residual standard error: 0.1704 on 55 degrees of freedom, multiple R-squared: 0.7113, Adjusted R-squared: 0.6903, F-statistic: 33.88 on 4 and 55 DF, *p* value: 2.985e-14

Notwithstanding these trends, it is noteworthy that there is no gradually or proportional decrease of incidence with distance increase to north region.

## Discussion

The main strength of our results resides in estimate of incidence density instead of cumulative incidence to consider the instability of population size from the beginning to the study end. Then, an additional advantage of incidence density is its accuracy due to the exact contribution of each animal in terms of animal-months at risk calculated at the denominator. That is not the case with cumulative incidence because the denominator is the mean of population size at start and the end of the study.

The cornerstone of results generated by the current study remains on accurate assessment of density incidence. There is a clear increasing of incidence density when regions are distant from coastal region. Yet, there is no proportional increase of density incidence the more the region is distant from the coast. These facts are contrasting with a Norwegian large-scale study on Human borreliosis, sheep and cattle anaplasmosis, substantiating a decreasing trend as far as the region is distant from the coast [14]. The explanation of this phenomenon, amongst multifactorial factors, is the sense of hygrometry gradient which is higher in coastal region and lower in distant ones. The more region is humid, the more ticks prevail, and their vectoral capacity is optimal. Considering ticks vectors factor, it encompasses ticks questing abundance, prevalence of pathogens in tick populations, number of tick bites per mammal host. Amongst others factors that impact incidence density, there are altitude, cattle and human population settlement [14], and health interventions [15]. Incidence density of babesiosis, whatever the aetiology (*Babesia bovis* or *Babesia bigemina*) and incidence density of anaplasmosis(Anaplasma marginale) were high in our study in comparison to the existing rare large-scale study [14]. This significant difference is due to laboratory confirmation in our study, whereas it was about clinical suspect cases in other studies. As consequences, it may exist a lot of false-positive cases in symptoms-based studies. The second reason in this difference is owing to diagnostic of many subclinical cases in our study. Of course, these subclinical cases are not able to be detected symptomatically. In the northern part of Kenya [16], lower incidences have been assessed because of the lower sensitivity of the smear test used to confirmed new cases of cattle babesiosis: 1.5 new case per 10,000 cattle-months in rural area and 5 new cases per 10,000 cattle-months in peri-urban area and anaplasmosis: 1.5 new cases per 1000 cattle-months in rural area, 1 new case per 100 cattle-months in peri-urban area. The same lower trend has been estimated with *Babesia bigemina* infection: 0–1 new case per 100 cattle-months; *Babesia bovis*: 0–3 new cases per 100 cattle-months; *Anaplasma marginale*: 1 new case per 1000 cattle-months in Nigeria [17] and north-eastern part of Tanzania (*Anaplasma marginale*: 0.08 new case per 100 cattle-months) [15].

Considering region altitude, it is noteworthy that it increases when incidence density of anaplasmosis decreases. There is exception for east region (altitude of 190 m) in which incidence density is smaller than south one (altitude from 10 to 15 m). This pattern has been shown by Mysterud *et al*. [14]. Interestingly, the contrary trend—altitude and incidence density increase together—is observed with both incidence density of *Babesia bovis* and *Babesia bigemina* infections. There is exception for east region in incidence density of *Babesia bovis* and *Babesia bigemina* infections. The other determinants could intervene to explain this discrepancy. Further studies are required to test hypothesis of cattle population density in the region and abundance of ticks’ population questing.

The positive correlation in our study for both diseases is very likely because tick’s species that transmit these pathogens are the same. Many studies on incidence of Babesiosis and Anaplasmosis, realised in Kenya [16], and Norway [14] substantiated the same correlation.

This is the first density incidence assessment of ticks borne diseases in west of Africa to the authors’ knowledge. Veterinarian authorities need to get accurate data on ticks borne diseases speed of spreading in cattle populations. Chief Veterinarian Officers will be enabled to anticipate early so relevant riposte or control. Incidence density remains a crucial epidemiological parameter in such requirements. The authors acknowledge that in an ideal situation, a national registry of suspects or confirmed cases each year, of OIE notifiable diseases including anaplasmosis, babesiosis, amongst 117 diseases would have to be available. These data would have permit us to refine or standardise our parameters’ estimates.

Amongst the limitations of the current study, there is absence of control measures. Our data reflect the natural strength of these diseases’ spread. Nevertheless, in reality, many farmers undertake actions such as the use of acaricides and drugs with more or less success. With such control measures leading to a competing events’ situation, we would have to use more complex statistical model that is Cox model for all event-specific hazards [18]. We should have included this factor in density incidence estimate study. Variables such as physiologic state, age, sex, and cattle breed level should have to be considered to demonstrate their possible impact as risk factor on incidence density.
